# Point of care testing evaluation of lateral flow immunoassay for diagnosis of cryptococcus meningitis in HIV-positive patients at an urban hospital in Nairobi, Kenya, 2017

**DOI:** 10.1186/s13104-019-4829-4

**Published:** 2019-12-05

**Authors:** Lawrence Kirimi Gitonga, Waqo Gufu Boru, Arthur Kwena, Marybeth Maritim, Joyce Wamicwe, James Ransom

**Affiliations:** 1 Field Epidemiology and Laboratory Training Program, Nairobi, Kenya; 2 Moi University, Eldoret, Kenya; 3 Ministry of Health, Nairobi, Kenya; 4 National AIDS and STI Control Program-Ministry of Health, Nairobi, Kenya; 5 University of Nairobi, Nairobi, Kenya; 6 Piret Partners Consulting, Washington, DC USA

**Keywords:** Point of care, Meningitis, Kenya, HIV

## Abstract

**Objectives:**

The objective of this study was to evaluate the performance of lateral flow immunoassay (LFA) against latex agglutination (LA), India ink and culture in point-of-care diagnosis of cryptococcus meningitis (CM). We conducted cross-sectional study among HIV-positive patients with suspected CM at Mbagathi Hospital, Nairobi, April–July 2017.

**Results:**

Of 124 capillary blood and serum and 99 cerebrospinal fluid (CSF) samples, LFA and LA had a concurrence on serum of 94.4%, kappa (0.88), sensitivity (100%) and specificity (91%). LFA and LA on CSF, was 97.9%, kappa (0.96), sensitivity (100%) and specificity (96%). LFA and India ink was 96.9%, kappa (0.94), sensitivity (100%) and specificity (94.1%). On CSF culture, concurrence was 72.7%, kappa (0.43), sensitivity (100%) and specificity (64%) and of LFA on capillary blood, serum and CSF was 100% with kappa (1.00), sensitivity and specificity of 100%.

## Introduction

Cryptococcus meningitis (CM) is a life-threatening opportunistic infection among HIV-infected persons [1]. In Africa, CM is the second leading cause of death in HIV-infected persons [2], with a case fatality rate (CFR) of up to 38% among outpatients and 81–100% among inpatients [3]. In Kenya, up to 33% of people with AIDS develop CM [4]. CM diagnosis usually occurs when meningitis is at an advanced stage and treatment is less effective [1, 5, 6].

Culture is the gold standard diagnostic method for CM, but it has poor sensitivity, requires approximately 100 µl of cerebral spinal fluid (CSF), technical expertise, and laboratory infrastructure [7, 8]. Microscopy requires laboratory infrastructure, and latex agglutination (LA) has sensitivity and specificity of > 99% and is less labour intensive than culture but also requires technical expertise and laboratory infrastructure. Culture and LA are not available in resource constrained settings, thus limiting their clinical utility [9].

Lateral flow immunoassay (LFA) is a point-of-care (POC) qualitative test to detect capsular polysaccharide antigens of *Cryptococcus species* complex (*Cryptococcus neoformans and Cryptococcus gattii*) [9–11]. LFA can use whole blood, serum, or CSF, is room temperature stable, has a rapid turnaround time of < 15 min, is simple to perform, and can be interpreted by personnel with minimal training [9].

Most studies evaluating LFA are focused on use of serum and CSF [7, 8, 12–14]. There are few data on use of LFA on capillary blood [15] or evaluation of LFA in Kenya. This study aimed to determine the agreement of test results from LFA on capillary blood, serum and CSF with those from LA on serum, CSF, India ink microscopy and culture on CSF.

## Main text

### Methods

#### Study design

We conducted a hospital-based cross-sectional study from April to July 2017.

#### Study site and population

The study was conducted at Mbagathi Hospital, a referral facility located in Nairobi County. The evaluation targeted patients ≥ 18 years scheduled for lumbar puncture (LP) and routine blood sample collection for CM diagnosis.

#### Inclusion criteria

LP requested by health care provider, availability of remnant serum and CSF (≥ 500 µl) after routine Cryptococcus LA or culture was performed on HIV-positive patients ≥ 18 years with the ability and willingness to provide informed consent.

#### Exclusion criteria

Patients involuntarily incarcerated in the hospital for psychiatric or physical illness, any patient/patient with a guardian who was deemed mentally unstable or unable to provide informed consent. Patients on any antifungal treatment and patients who were having repeat LPs.

#### Sample size assumptions and calculation

The sample size of 125 participants was calculated using Fisher’s formulae, assuming the expected proportion of agreement between LFA and other methodologies (p) was 97.7% and the precision (P) of 3% [16].

#### Sampling methods

We reviewed records for 6 months at Mbagathi Hospital and obtained an average of 95 cases of suspected CM in a month. To achieve a sample size of 125 patients using the estimated sampling frame of 285 within a period of 3 months, every second HIV-positive patient ≥ 18 years suspected of CM and scheduled for routine LP and blood collection for CM diagnosis was enrolled after giving written consent to obtain an additional capillary blood and use of the remnant CSF and serum for evaluation of LFA.

#### Data collection

**Fig. 1:**
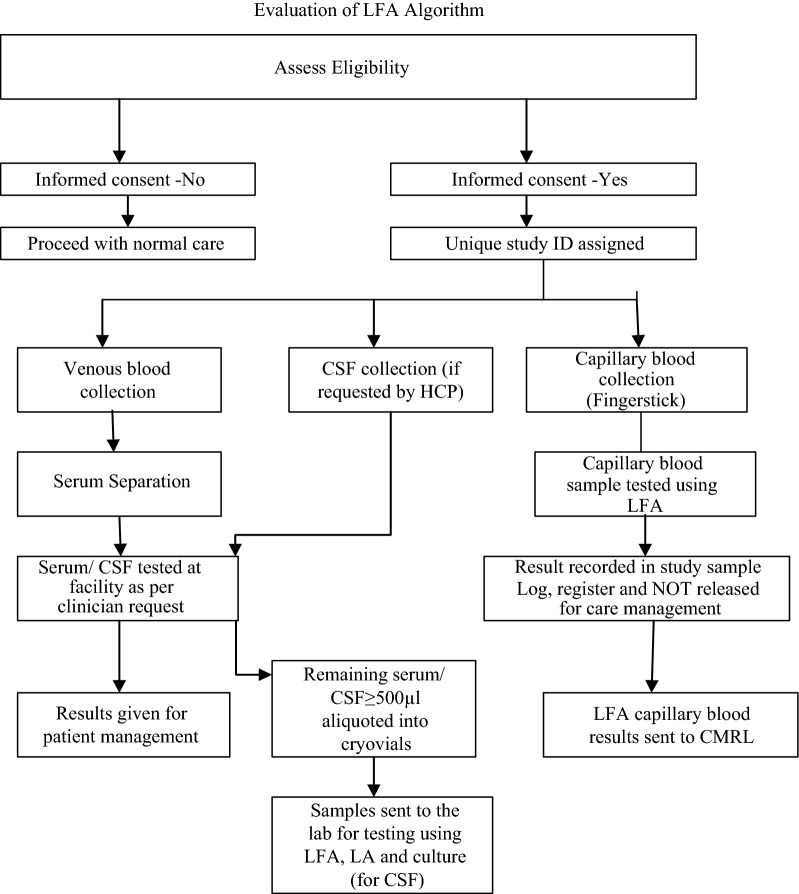
Evaluation of LFA algorithm

The attending laboratory technologist collected blood samples from patients as part of routine testing requested by the clinician. Serum was centrifuged and separated for LA and LFA assays. CSF samples were collected by LP and centrifuged. The supernatant was used for LFA and LA assays and the pellet for culture. Sera were tested by LA, and CSF (where available) was tested by India ink microscopy and culture for clinical management of the patient. Leftover sera and CSF samples were used for the laboratory evaluation. A minimum of 500 µl of the remaining sample was aliquoted into 1.8 ml cryogenic vials. The samples were stored at 4 °C for a maximum of 72 h or at − 20 °C awaiting transportation to the Central Microbiology Reference Laboratory (CMRL). Additionally, a non-routine finger prick capillary blood sample was requested from all enrolled patients. Using standardized lancets and micro capillary tubes, ~ 50 µl of blood was transferred into micro centrifuge tubes containing LFA specimen diluent. The test was performed at the sample collection site per manufacturer instructions. Sera and CSF sample processing were done at CMRL per laboratory standard operating procedures and manufacturer instructions (Fig. 1).

#### Data management and analysis

Data on the test type and test results in different sample types were entered and cleaned in MS-Excel version 2013. Statistics were calculated using GraphPad QuickCalcs 2017 (GraphPad Software, Inc., La Jolla, CA) with categorical data analysis to assess sensitivity, specificity, predictive values, confidence intervals (CIs) of proportion, overall percent agreement, and kappa (*k*) coefficients of India ink, LA, LFA and culture in sera, CSF and capillary blood. Interpretation of *k* was per standard guidelines [17].

### Results

**Fig. 2:**
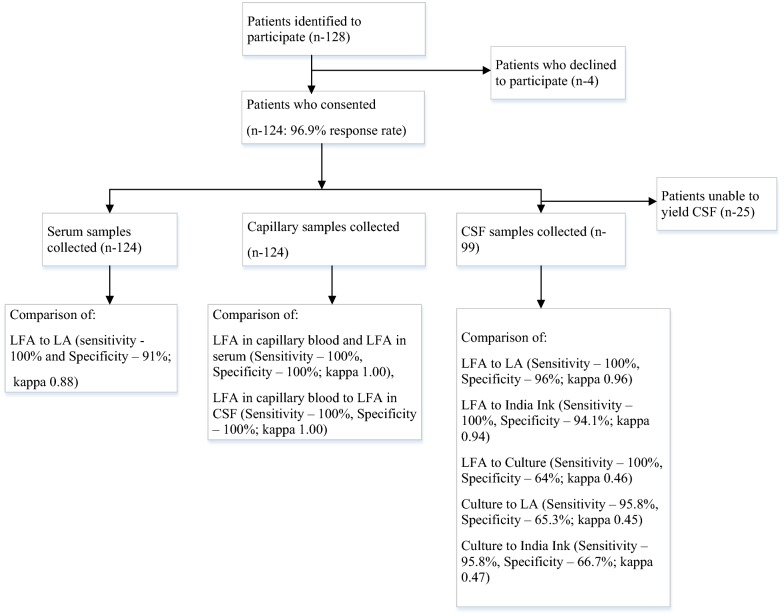
Outcomes algorithm

Figure 2 outlines the results of the comparison studies. Out of 128 persons suspected of CM, 124 were enrolled in the study. A total of 124 capillary blood and serum samples, and 99 CSF samples, were analysed. Twenty-five patients were not able to yield CSF sample. Comparing LFA to LA on sera, the sensitivity and specificity were 100% (95% CI 92.3–100) and 91% (95% CI 82.6–95.6) respectively, PPV and NPV at 86.8% and 100% with a total agreement of 94.4%, and a kappa of 0.88 (95% CI 0.80–0.97). LFA to LA on CSF, the sensitivity and specificity were 100% (95% CI 92.7–100) and 96% (95% CI 86.5–98.9), PPV and NPV at 96.1 and 100 with a total agreement of 98% and a kappa-value of 0.96 (95% CI 0.90–1.00).

Comparison of LFA to India ink (microscopy) using CSF, the sensitivity and specificity were 100% (95% CI 92.6–100) and 94.1% (95% CI 84.1–97.9), PPV and NPV at 94.1% and 100% with a total agreement of 97% and a kappa-value of 0.94 (95% CI 0.87–1.00). On comparison of LFA to culture on CSF, the sensitivity and specificity were 100% (95% CI 86.2–100) and 64% (95% CI 52.7–73.9), PPV and NPV at 47.6% and 100% with a total agreement of 72.7% and a kappa-value of 0.46 (95% CI 0.32–0.61).

Comparison of culture to LA using CSF, the sensitivity and specificity were 95.8 (95% CI 79.8–99.3) and 65.3 (95% CI 54.1–75.1) with a total agreement of 72% and a kappa value of 0.45 (95% CI 0.30–0.60). On comparison to India ink, the sensitivity and specificity were 95.9% (95% CI 79.8–99.3) and 66.7% (95% CI 55.4–76.3) with a total agreement of 73% and a kappa value of 0.47 (95% CI 0.31–0.62).

Comparison of LFA on capillary blood to LFA on sera, the sensitivity and specificity were 100%, PPV and NPV with a total agreement of 100%, and a kappa-value of 1.00 (95% CI 1.00–1.00). LFA on capillary blood was compared to LFA on CSF, the sensitivity, specificity and predictive values were all 100% with a total agreement of 100% and a kappa-value of 1.00 (95% CI 1.00–1.00).

### Conclusion and recommendation

#### Conclusion

Our results show high agreement between LFA, LA and India ink in different samples and a perfect agreement between LFA in different samples. The high agreement shows that LFA is a reliable POC diagnostic test. The results on individual tests show that there was almost perfect agreement between LFA and LA on CSF and serum. The test demonstrated high level of sensitivity and specificity of LFA compared to LA on sera and CSF. These findings are consistent with similar studies conducted in South Africa and USA that show high sensitivity using CSF and serum [12, 18]. Comparable results were reported in a study on multisite validation of cryptococcal antigen lateral flow assay in Uganda and South Africa [7]. The strong agreement between the LFA and LA tests is an indicator that LFA test on whole blood, CSF and serum is as good as LA test on sera and CSF.

The findings from comparison of LFA to India ink microscopy using CSF demonstrated high sensitivity, specificity, and predictive values. This is in contrast to the findings from the expert opinion and other studies that documented lower sensitivity and NPV for CSF microscopy against LFA [7, 9]. The India ink microscopy requires laboratory infrastructure, dependent on fungal concentration and is highly operator dependent rather than the test performance.

On comparison of LFA to culture using CSF, there was high sensitivity, low specificity, and moderate agreement with a weak kappa value. The findings were consistent with other studies that documented high sensitivity and low specificity [14, 19]. The findings on high sensitivity, low specificity and a weak kappa value were similarly demonstrated when CSF culture was compared to LA and India ink using CSF. However, other studies documented low sensitivity in CSF culture when compared against other diagnostic tests [7–9].

LFA on capillary blood was compared with LFA on serum and CSF. The LFA results on capillary blood had an ideal concordance with LFA serum and CSF results. LFA had a very high positive and negative predictive values both on serum and CSF, a characteristic that makes it good for an accurate diagnosis of cryptococcal meningitis. The high sensitivity and specificity of the test and its ability to be easily performed at the bedside and giving accurate results rapidly allows for prompt and timely initiation of treatment [9]. The findings were comparable with a similar study on evaluation of LFA using serum, CSF and capillary blood in Uganda [15, 18, 20].

#### Recommendation

The evidence of the perfect agreement between LFA on capillary blood, serum and CSF, high sensitivity and specificity, ease of performance, along with rapid results may indicate LFA using capillary blood POC test as the method of choice for CM diagnosis. Our results show that LFA meets World Health Organization assured criteria for POC diagnostic tests in resource-limited settings. Therefore, we recommend use of LFA test as a POC test in resource limited settings for the diagnosis of CM.

## Limitation of the study

The limitations of this study include participants not yielding CSF sample due to dry taps, thus reducing the CSF samples that were analysed. The difference on CSF samples analysed had no major implications to the overall evaluation since there were more than one sample type used in the evaluation. Capillary blood could only be used on LFA test thus, there was no uniform use of the sample type across other testing procedures.

## Data Availability

All data related to this study are available upon request.

## Abbreviations

AFENET: African Field Epidemiology Network
CFR: case fatality rate
CI: confidence interval
CM: cryptococcal meningitis
CMRL: Central Microbiology Reference Lab
CSF: cerebrospinal fluid
LA: latex agglutination
LFA: lateral flow immunoassay
LP: lumbar puncture
NPV: negative predictive value
POC: point of care
PPV: positive predictive value
